# Impact of perineal healing on oncological outcome following surgery for squamous cell carcinoma of the anus

**DOI:** 10.1111/codi.70130

**Published:** 2025-06-08

**Authors:** Naseer Baloch, A. Kiasat, Mikael Machado, Jonas Nygren, Per J. Nilsson

**Affiliations:** ^1^ Division of Coloproctology, Department of Pelvic Cancer Karolinska University Hospital Stockholm Sweden; ^2^ Department of Molecular Medicine and Surgery Karolinska Institutet Stockholm Sweden; ^3^ Department of Clinical Sciences at Danderyds Hospital Karolinska Institutet Stockholm Sweden; ^4^ Department of Surgery Ersta Hospital Stockholm Sweden

**Keywords:** anal cancer, cohort study, recurrence, surgery, survival

## Abstract

**Aim:**

Perineal wound complications are common following salvage surgery in anal cancer. It is not fully known if healing disorders have an impact on oncological outcomes. The aim of this study is to investigate survival in relation to perineal healing status at 3 months after surgery.

**Method:**

A retrospective cohort study including all patients with squamous cell anal cancer operated on between January 2005 and June 2020 in Stockholm, Sweden was undertaken. Data collection was by registers and supplemented by chart review. All patients were followed until death or 31 December 2020. Perineal healing status at 3 months was used as a landmark. Association between healing status and recurrence free (RFS) and overall survival (OS) were evaluated using the Cox proportional hazard regression model.

**Results:**

The final study population comprised 122 patients (78 women). At the landmark date, 73 patients (60%) had a healed perineum and 49 (40%) an unhealed perineum. The R0 resection rate was 92% (112 patients). Follow‐up ranged between 6 and 185 months. Five‐year OS and RFS for all patients was 62% and 56%, respectively. OS was 71% vs. 48% and RFS was 65% vs. 43% for patients with healed versus unhealed wound at the landmark date, respectively. Healing status was significantly associated with OS in univariable analysis (HR 0.52, *p* = 0.021) and RFS showed a trend (*p* = 0.054) in the same direction. In a multivariable analysis adjusting for age, gender and T‐stage a statistically significant difference was found concerning OS (HR = 0.47, 95% CI 0.26–0.85) and a trend in the same direction for RFS (HR = 0.60, 95% CI 0.34–1.06).

**Conclusion:**

The hypothesis that an unhealed perineal wound following salvage surgery for patients with squamous cell anal carcinoma not only constitutes a surgical but also an oncological problem is strengthened by this study.

**What does this paper add to the literature?**

To our knowledge this is the first paper to review the association between healing disorder and oncological outcome following salvage abdominoperineal excision for squamous cell cancer of the anus. It may indicate a negative association between an unhealed perineal wound and oncological outcome.

## INTRODUCTION

Although most patients with squamous cell carcinoma of the anus (SCCA) obtain a complete response after chemoradiotherapy, up to 30% may eventually need surgery [1, 2, 3]. Surgery, often referred to as ‘salvage surgery’, usually consists of an abdominoperineal excision (APE), but more extensive operations such as posterior pelvic exenteration (PPE) or total pelvic exenteration (TPE) may be necessary to achieve a clear resection margin. Some patients undergo surgery for other reasons, for example previous radiotherapy to the pelvis. Salvage surgery can offer durable local control and survival, with 5‐year survival rates reported to be between 33% and 78% [4, 5, 6, 7, 8, 9, 10].

Surgery for anal cancer is associated with major perineal complications such as wound infection, dehiscence, fistula formation, abscess formation with chronic inflammation and delayed healing times extending up to a year after surgery [4, 5, 11, 12, 13, 14]. Results have improved with the introduction of perineal reconstruction with myocutaneous flaps, but the problem is not completely solved. A significant proportion of patients still have an unhealed perineal wound during a prolonged postoperative period [15, 16].

Virchow is credited for an early observation that tumours often develop where chronic inflammation is present [17]. Dvorak in his 1986 review on links between inflammation and cancer showed that wound healing and tumour stroma share many properties such as DNA damage, negative regulation of p53, apoptosis evasion and angiogenesis [18] There is evidence of varying quality indicating an association between inflammation and prostate cancer, lung cancer and colorectal cancer [19, 20]. Similarly, data show a significant association between colorectal anastomotic leakage and a higher local recurrence rate, reduced disease‐free survival and reduced long‐term cancer‐specific survival [21, 22]. It may therefore be reasonable to hypothesize that patients with a prolonged period with an unhealed perineal wound following salvage surgery for SCCA could have a poorer oncological outcome than patients with a healed perineum after surgery.

This study aims to investigate any potential association between an unhealed perineal wound and oncological outcomes such as recurrence and survival.

## METHOD

This is a retrospective observational study including all patients who underwent salvage surgery for SCCA in Stockholm, Sweden between January 2005 and June 2020. The study was approved by the regional Swedish ethical review authority (Dnr: 2017/1894‐31/1). This study is reported in accordance with the STROBE guidelines.

Patients with SCCA operated on during the study period were identified through a regionally maintained register on all anal cancer patients. All operations were at either Ersta Hospital or Karolinska University Hospital and in‐house registers on operations performed from both hospitals were searched to find additional patients. Surgical outcomes with a focus on perineal healing for part of this cohort have previously been reported [11].

In brief, characteristics for each patient were retrieved retrospectively from patient charts and from the ERAS interactive audit system (erassociety.org). In all patients the diagnosis of SCCA was confirmed by biopsy. Data extracted from patient charts were age, gender, initial tumour stage according to TNM version 6 between 2005 and 2009 and version 7 from 2010 and onward [23, 24] and fractionation and total dose of radiotherapy. In addition, time from conclusion of radiotherapy to surgery and operative details including type of operation, i.e. APE, PPE or TPE, and type of perineal reconstruction were retrieved. Tumours were categorized as residual if the tumour was detected within 6 months of conclusion of oncological treatment and as recurrent if detected later than 6 months after termination of radiotherapy. Patients who had previously undergone APE and presented with a recurrence were classified as having a re‐recurrence. Specimen histopathology and resection status (R0/R1) were recorded.

In the early part of the study period patients with tumours larger than 4 cm and/or node positive were treated with platinum‐based induction chemotherapy. Radiotherapy consisted of 2 Gy fractions to 46 Gy when a response evaluation was performed, and in case of a good response a boost up to 64 Gy was delivered. Volumetric modulated arc therapy was introduced 2008. In 2017 a national care programme was introduced in which radiotherapy is usually combined with chemotherapy (mitomycin C with fluorouracil or capecitabin). Patients with early tumour (T1N0M0) were prescribed a radiotherapy dose between 45 and 50 Gy, so‐called regimen A, those with primary tumour size <4 cm (T2–T3N0) were prescribed a radiotherapy dose of 50–55 Gy (regimen B) and patients with a tumour size ≥4 cm or with pathological lymph nodes were prescribed a radiotherapy dose of 50–60 Gy depending on the size of metastases (regimen C) (Figure S1). Follow‐up was according to the institutional clinical routine.

Patients in whom a residual or recurrent tumour was detected were assessed at a multidisciplinary tumour board prior to surgery. Preoperative assessment included MRI of the pelvis and thoracoabdominal CT scanning in all patients. Although not mandatory according to national guidelines before 2017, most patients also underwent preoperative positron emission tomography/CT to exclude distant metastases. During the study period no national guidelines existed concerning follow‐up after surgery, but generally the first postoperative follow‐up was after 4–6 weeks and then every 6 months during the first 2 years, followed by 1‐year intervals for a total of 5 years. During follow‐up after surgery, no radiological examinations were mandatory although most patients underwent CT scans on an annual basis.

For all patients, date of detection of recurrence, locoregional, distant or combined, and date of death was registered. For the purpose of this study, patients were divided into two groups: patients with a fully healed or with an unhealed perineal wound at 3 months after surgery. The cut‐off of 3 months was selected based on our previous findings that despite significant differences in healing rate at 3 months, most patients had healed wounds 1 year after surgery [11]. If death or recurrence occurred within 3 months after surgery the patient was excluded from further analysis. All patients were followed until 31 December 2020, or death.

### Statistical methods

For the survival analysis regarding recurrence free survival (RFS) and overall survival (OS) a landmark was set to 3 months after the date of surgery, corresponding to time when perineal healing was assessed. Time to failure was calculated from the landmark date to the date of disease recurrence or date of death, whichever came first. For event‐free patients time was calculated from the date of the landmark to the common closing date of 31 December 2020. Time to death was calculated from the date of the landmark to the date of death or to the common closing date.

The effect of covariates on time to failure or time to death was graphically displayed using Kaplan–Meier curves. Proportional hazards regression was used in the univariate and multivariate analysis of time to failure or death. The assumption of proportional hazards was tested using Schoenfeld residuals. In the multivariate analysis age (≤65 vs. >65 years), sex and stage (T1–T2 vs. T3–T4) were included in the models as well as healing status. Results from the regression models are presented as hazard ratio (HR), together with 95% CI and Wald *p*‐values.

The statistical software Stata version 16 was used in all statistical analyses.

## RESULTS

**FIGURE 1:**
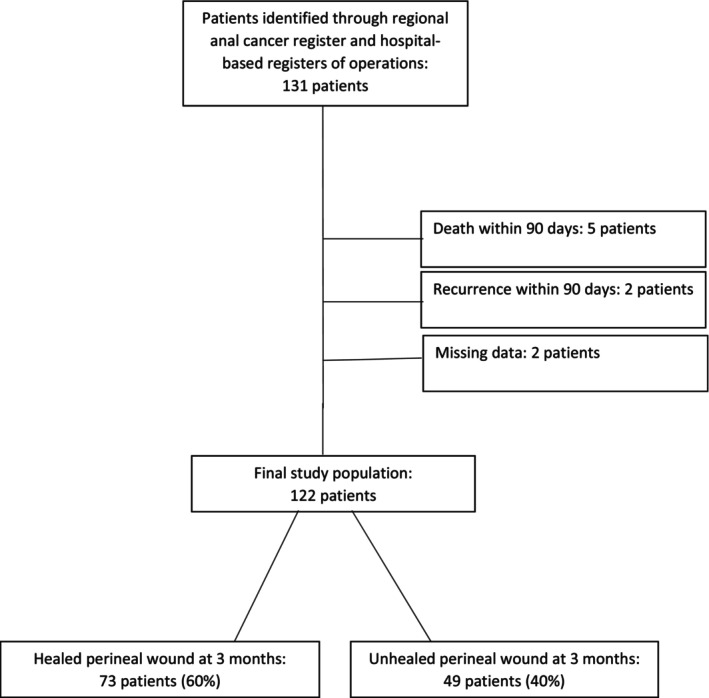
Study population.

Between 2005 and 2020, 131 consecutive patients underwent surgery for SCCA in Stockholm. Within 3 months of surgery five patients had died and two had a recurrence, and these were excluded alongside two patients with missing data. One patient underwent two operations for SCCA during the study period, initially for a residual tumour and then 2 years later for a re‐recurrence; only the initial operation was included in the analysis. In Figure 1, a flow chart of the study population eventually consisting of 122 patients is presented. Among these patients, 49 (40%) had an unhealed perineal wound and 73 (60%) were healed at 3 months after surgery.

Table 1 presents the initial tumour and treatment characteristics as well as patient characteristics at the time of surgery and surgical details. About two‐thirds of patients were classified as having a residual tumour, and this is reflected in the large proportion of patients having surgery after 46 Gy. In 27 operated patients, 21 of whom had received up to 46 Gy, no remaining tumour cells could be detected in the specimen histopathology. R0 resection was achieved in 112 patients (92%). As reported previously younger age and reconstruction with a vertical rectus abdominis myocutaneous flap were associated with better perineal healing [11].

**TABLE 1:** Patient and treatment characteristics.

	All patients	Healed	Unhealed
Number of patients	122	73	49
Age (years), median (range)	67 (34–89)	61 (34–86)	67 (37–89)
Age (years)			
≤65	72	52	20
>65	50	21	29
Gender (M:F)	44:78	26:47	18:31
Initial tumour stage			
cT1–2	56	34	22
cT3–4	62	35	27
Re‐recurrent	3	3	0
Missing	1	1	0
cN0	62	38	24
cN+	58	33	25
Missing	2	2	0
M0	115	71	44
M1	6	1	5
Missing	1	1	0
Initial treatment			
No RT	4	3	1
Previous RT	4	3	1
RT ≤ 46 Gy, ± chemo	59	37	22
RT > 46 Gy, ± chemo	54	29	25
Missing	1	1	0
Operation type			
APE	104	62	42
PPE	11	8	3
TPE	7	3	4
Reconstruction method			
Primary closure	42	19	23
VRAM flap	34	30	4
Gluteus flap	46	24	22
Resection margin status			
R0	112	67	45
R1	10	6	4
Histopathology			
pT0	28	16	12
pT1–T4	93	56	37
Missing	1	1	0
pN0	107	66	41
pN+	14	6	8
Missing	1	1	0

Abbreviations: APE, abdominoperineal excision; F, female; M, male; PPE, posterior pelvic exenteration; RT, radiotherapy; TPE, total pelvic exenteration; VRAM, vertical rectus abdominis myocutaneous.

**FIGURE 2:**
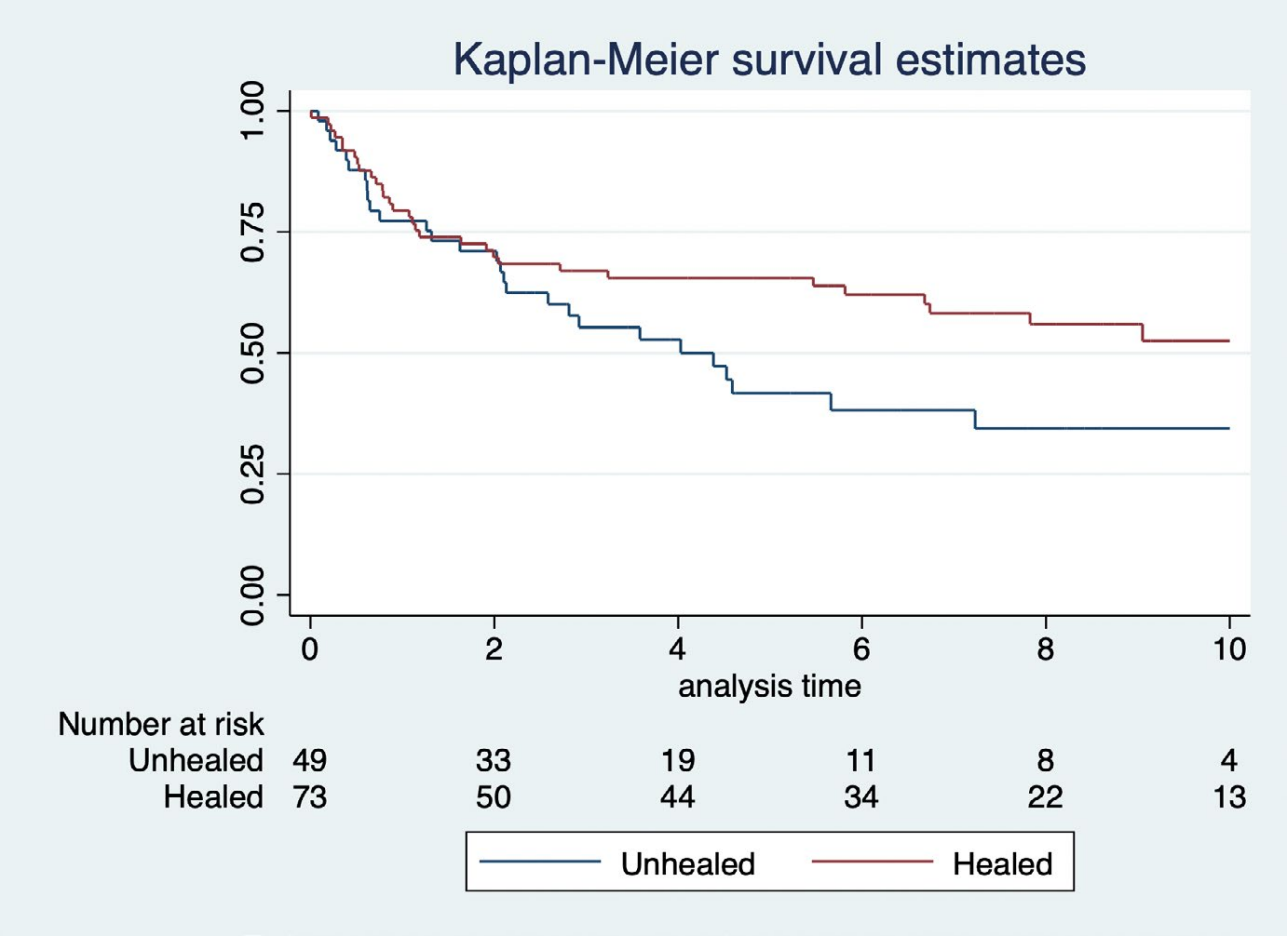
Overall survival in relation to healing status at 3 months after surgery (*p* = 0.016, log rank).

**FIGURE 3:**
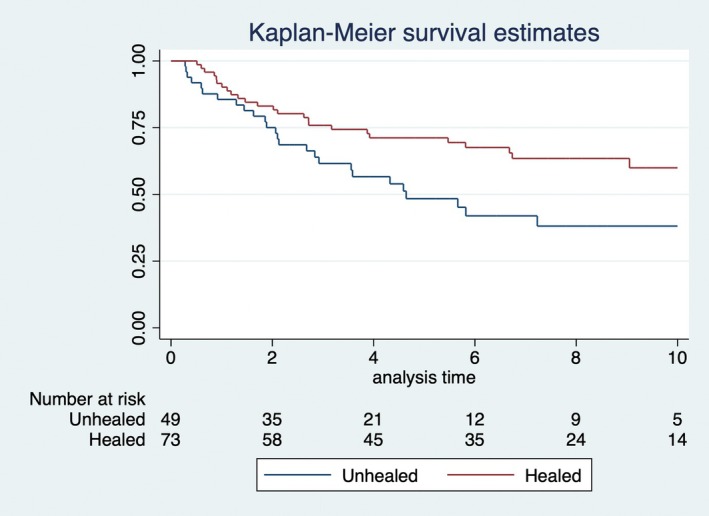
Recurrence‐free survival in relation to healing at 3 months after surgery (*p* = 0.052, log rank).

Table 2 presents the absolute numbers for recurrence and survival. OS and RFS at 5 years for all 122 patients were 62% and 56%, respectively. OS was statistically significantly worse for patients with an unhealed wound at 3 months (Figure 2). In addition, RFS was numerically poorer for patients with an unhealed perineal wound at 3 months after surgery (Figure 3). In the Cox proportional hazards regression analyses healing status was statistically significantly associated with OS in the univariable and multivariable analysis when adjusted for age, sex and T‐stage (Table 3). Although not statistically significantly associated, there was a trend (*p* = 0.054) for healing status in relation to RFS in the univariable model (Table 3).

**TABLE 2:** Oncological outcome following salvage surgery for anal cancer.

	All patients	Healed	Unhealed
	(*n =* 122)	(*n* = 73)	(*n* = 49)
Recurrence after operation			
Yes	38	23	15
No	84	50	34
Type of recurrence			
Locoregional only	25	15	10
Local + distant	4	3	1
Distant metastasis only	9	5	4
Deceased			
No	69	46	23
Yes	53	27	26
Missing	0	0	0

**TABLE 3:** Cox analyses on survival.

	OS (univariable)	OS (multivariable)	RFS (univariable)	RFS (multivariable)
Healing status				
Unhealed	1.0 (ref.)	1.0 (ref.)	1.0 (ref.)	1.0 (ref.)
Healed	0.51 (0.20–0.89)	0.47 (0.26–0.85)	0.60 (0.36–1.01)	0.60 (0.34–1.06)
Age				
≤65 years	1.0 (ref.)	1.0 (ref.)	1.0 (ref.)	1.0 (ref.)
>65 years	0.92 (0.52–1.62)	0.72 (0.40–1.32)	1.06 (0.63–1.77)	0.87 (0.49–1.54)
Gender				
Male	1.0 (ref.)	1.0 (ref.)	1.0 (ref.)	1.0 (ref.)
Female	0.68 (0.38–1.22)	0.75 (0.36–1.36)	0.66 (0.38–1.21)	0.72 (0.43–1.21)
T‐stage				
T1–2	1.0 (ref.)	1.0 (ref.)	1.0 (ref.)	1.0 (ref.)
T3–4	1.56 (0.89–2.74)	1.50 (0.85–2.65)	1.19 (0.71–1.99)	1.17 (0.68–1.97)

## DISCUSSION

Perineal wound healing complications following surgery for anal cancer are a well‐known clinical problem [5, 25, 26]. Prolonged healing times are not only resource demanding for healthcare systems but can also inflict pain, odour and suffering for the patient. Therefore, pre‐, intra‐ and postoperative measures to facilitate perineal wound healing are of great importance. We have previously reported that a vertical rectus abdominis myocutaneous flap appears to yield improved results compared with primary closure or a gluteus flap [11]. The present study aims to investigate whether an unhealed perineal wound may also be associated with a worse oncological outcome in order to further underline the importance of perineal wound management following surgery for anal cancer. The biological wound healing process is expected to be complete at 3 months, hence a landmark was set to 3 months after the date of surgery.

Despite reporting on a 15‐year series of consecutive operations for anal cancer, only 122 evaluable patients are reported on, which reflects the rarity not only of anal cancer but more so of surgery for anal cancer. In recent reports, only about 8% of anal cancer patients become candidates for major surgery [27]. The limited number of patients available for analysis also puts restraints on possibilities for statistical analysis. In this report we focus on OS and RFS and adjust only for age, gender and T‐stage. There are other parameters such as N‐stage, p16‐status, type and dose of (chemo)radiotherapy and time from radiotherapy to surgery that may be of interest but would need a larger dataset in order to be analysed in a meaningful manner. Borg et al. recently published results from a cohort of 98 patients who underwent salvage surgery for SCCA [4]. Contrary to this report, they could not find an association between complication and survival (HR 1.2). This difference in results is difficult to fully explain but may be an effect of a type‐II error because of small sample size.

Although there are several limitations to this study, we were able to show that an unhealed perineal wound at 3 months after surgery appears to have an impact on both OS and RFS. The graphical illustrations (Figures 2 and 3) clearly give an indication that healing status may be associated with a poorer oncological outcome. Younger age could be expected to be associated with a better healing outcome, which could not be shown in this study; however, the Cox analyses indicate that there is statistically significant association between an unhealed perineal wound and inferior OS.

Besides the primary hypothesis leading to the present study we were able to demonstrate a R0 resection rate of 92% and an OS for the entire group of 112 patients of 62%, indicating that the surgery performed was in line with international standards. In conclusion, the hypothesis that perineal complications following APE for anal cancer not only constitute a surgical problem but may also constitute an oncological concern can be considered strengthened by this study.

## AUTHOR CONTRIBUTIONS

**Naseer Baloch:** Conceptualization; investigation; writing – original draft; software; formal analysis; methodology; data curation; writing – review and editing; visualization; project administration; validation. **A. Kiasat:** Software; validation; data curation; writing – review and editing. **Mikael Machado:** Conceptualization; writing – review and editing; data curation; formal analysis. **Jonas Nygren:** Conceptualization; investigation; writing – review and editing; formal analysis; data curation. **Per J. Nilsson:** Conceptualization; investigation; funding acquisition; writing – original draft; methodology; validation; visualization; writing – review and editing; software; formal analysis; project administration; data curation; supervision; resources.

## FUNDING INFORMATION

Grant support from the Foundation for Surgical Cooperation, Stockholm, Sweden.

## CONFLICT OF INTEREST STATEMENT

None.

## ETHICS STATEMENT

This research was approved by the Swedish Ethical Review Authority (Dnr: 2023‐05304‐02).

## Supporting information


Data S1.

## Data Availability

The data that support the findings of this study are available on request from the corresponding author. The data are not publicly available due to privacy or ethical restrictions.
