# Supplementary material for: Impact of perineal healing on oncological outcome following surgery for squamous cell carcinoma of the anus
Source: Colorectal Dis. 2025 Jun 8;27(6):e70130. doi: 10.1111/codi.70130 (PMC12146502; doi:10.1111/codi.70130)
Supplement: Supplementary file 1 — Data S1. [file CODI-27-0-s001.docx]

Supplementary Figure 1

Overview of oncological treatment regimens over time

Tumour >4 cm
AND/OR
Node positive

Tumour <4 cm AND
Node negative

Legend to Figure:

EBRT: external beam radiotherapy. *: From 2005 to 2008 EBRT delivered with 3D conformal technique and thereafter with volumetric modulated arc therapy (VMAT). 5FU: 5-fluoruracil. MMC: Mitomycin C

2017

EBRT 58 Gy
MMC/5FU x2 concomitant

EBRT 55 Gy
MMC/5FU x1 concomitant

T1-T2
Tumour <4 cm
AND N0

T2-T4
Tumour >4 cm
AND/OR N+

Boost EBRT* to 64 Gy

Boost EBRT* to 64 Gy

Assessment

Assessment

EBRT* 46 Gy

EBRT* 46 Gy

Induction chemotherapy
x3
Cisplatinum/5FU
